# Key determinants of “Serious Circumstances” in awarding punitive damages for intellectual property infringement: Evidence from Chinese Judicial Judgments

**DOI:** 10.1371/journal.pone.0340113

**Published:** 2026-01-21

**Authors:** Kaijie You

**Affiliations:** Tan Siu Lin Business School(School of Intellectual Property), Quanzhou Normal University, Quanzhou, Fujian, China; Universiti Kebangsaan Malaysia, MALAYSIA

## Abstract

To prevent the over-extension of punitive damages in private law, as has occurred in some common law jurisdictions, China requires the presence of “serious circumstances” as a prerequisite for applying punitive damages in intellectual property infringement cases. However, this requirement still lacks a clear theoretical framework and concrete identification elements. This study illustrates China’s approach by analyzing judicial decisions, and reveals: (1) sixteen elements used by judges to assess the seriousness of intellectual property infringement, which can be consolidated into three key categories: subjective malevolence, degree of conduct, and resulting damage; (2) differences in judicial emphasis across types of intellectual property, with copyright cases focusing on subjective malevolence and industrial property cases emphasizing scale and duration of infringement; (3)cases involving significant social harm are more likely to result in higher punitive damages multipliers; and(4) judges take into account not only the severity of infringement but also the goals and functions of punitive damages.

## Introduction

Punitive damages refer to monetary compensation awarded to the infringed party in excess of actual losses. They serve both compensatory and deterrent functions by punishing wrongful conduct and discouraging future infringements [1]. In intellectual property disputes, the intangible nature of the subject matter and the complexity of market valuation often result in “difficulty of proof and low compensation”, limiting the effectiveness of traditional remedies. To strengthen protection, common law countries incorporated punitive damages for malicious infringement as early as the late eighteenth century. In contrast, continental law countries traditionally rejected punitive damages, viewing them as blurring the boundary between public and private law and contravening the compensatory principle of civil liability [2]. In recent years, however, scholars in continental law systems have softened this stance, increasingly recognizing the value of punitive damages in fields such as intellectual property [3]. While rooted in the continental law tradition, China has selectively drawn from the experience of common law jurisdictions. To effectively curb infringement, further stimulate innovation, and maintain public order, China established a comprehensive punitive damages regime for intellectual property in its 2020 Civil Code [4]. This development marks the first systematic introduction of punitive damages for intellectual property within a continental law framework.

Given the punitive character of this remedy, Chinese judges exercise caution in its application. In intellectual property disputes, punitive damages may only be awarded if the objective requirement of “serious circumstances” is satisfied [5]. Yet no unified framework or detailed standard currently exists—domestically or internationally—for identifying such circumstances. As a result, judicial practice lacks consistency, and litigants cannot reliably predict outcomes. Establishing a structured framework and detailed criteria for “serious circumstances” would therefore enhance judicial predictability and fairness in applying punitive damages.

China’s practice demonstrates a dual approach: bold in establishing a comprehensive punitive damages system for intellectual property, yet prudent in judicial application. As a continental law country, China reflects both innovation in legislative design and restraint in adjudication, aiming to balance deterrence with fairness in private law.

This study utilized the case analysis method to extract the elements of serious circumstances from the intellectual property infringement judgments in China involving punitive damages, and to analyze which elements constitute the key determinants of serious circumstances in various intellectual property cases. This study developed an overall framework and specific details for determining serious circumstances justifying punitive damages in intellectual property infringement cases.

This study sought to develop an overall framework and specific criteria for identifying serious circumstances, with the goal of offering more predictable guidance for judges and parties. Through the analysis of intellectual property infringement cases in China, this study illustrates how the punitive compensation system can be integrated into the civil law framework and applied in a measured and balanced manner within the private rights domain of intellectual property.

### Literature review

Current research on punitive damages for intellectual property infringement has primarily focused on their legitimacy and the conditions for application, along with the determination of the base and multiplier of damages.The legitimacy of applying punitive damages to intellectual property infringement, once controversial, has now gained broader acceptance. Traditionally, continental law scholars resisted introducing punitive damages into intellectual property law, regarding it as a matter of public law. More recently, however, their stance has softened [6], with acceptance of punitive damages in areas such as intellectual property rights and anti-competitive behaviour [3]. Many scholars argue that applying punitive damages in intellectual property aligns with both the principle of justice and the economic principle of cost-benefit analysis [7,8]. Punitive damages serve multiple functions, with compensation [9], punishment [10], and deterrence [11] being the most central. Additional functions include maintaining social balance [12]and encouraging right holders to pursue litigation [13]. Because intellectual property infringement often involves abstract objects, technical complexity, concealment, evidentiary difficulties, low compensation, and repeated malicious conduct [14,15], it is particularly challenging to evaluate losses accurately. These limitations weaken the compensatory and preventive effects of traditional tort remedies [16]. In situations where traditional tort remedies fail to adequately address intellectual property infringement, the introduction of punitive damages can effectively compensate right holders for their losses, curb future infringements, and stimulate innovation [7,15,17]. With their distinct socio-legal character and capacity to promote social justice, punitive damages can also find a meaningful role within the civil law (private law) system [18]. Thus, their application to intellectual property is both legitimate and necessary.

In common law jurisdictions, legislation rarely specifies the conditions for applying punitive damages in intellectual property cases; these conditions are shaped through judicial practice. As early as *Seymour v. McCormick* patent infringement case, U.S. judges emphasized intentional infringement as the standard [19]. Over time, courts identified three forms of intentional infringement: wilful copying of another’s invention, circumvention of reasonable investigation into patent validity, and misconduct during litigation [20]. In *re Seagate Tech., LLC*, the court introduced a “uniform subjective and objective standard”, requiring proof that the defendant’s conduct posed an objectively substantial risk of continued infringement and that the defendant was subjectively aware of this risk [21]. Later, in *Stryker Corp. v. Zimmer, Inc*. [22] and *Halo Elecs., Inc. v. Pulse Elecs., Inc.* [23], the courts rejected this stringent standard, holding that requiring both elements allowed defendants to evade liability. The “and” in the standard should be replaced by “or”. U.S. judges now often refer to nine subjective and objective factors when deciding whether punitive damages are appropriate [24].

Canada likewise does not explicitly stipulate the conditions for applying punitive damages in legislation. Judges have generally held that punitive damages are an unusual remedy that applies only when the infringer has committed a more egregious infringement [25], and the infringer’s conduct must be either a reprehensible act or repeated infringement [26]. Although punitive damages originated in the UK, their application there is stricter than in the United States, limited primarily to malicious or serious infringements. British courts consider whether the infringement was intentional, whether it was repeated, and the duration and scope of the conduct [27].Overall, while common law countries do not explicitly codify “serious circumstances” as a condition, their jurisprudence emphasises the role of wilfulness and bad faith.

As civil law countries generally reject the application of a punitive damages system in the field of private law, it is difficult to identify the assessment criteria of “serious circumstances” when applying such a system to intellectual property in continental law countries. In 2020, China explicitly stipulated in the Civil Code that the application of the intellectual property punitive damages system must satisfy the requirements of “intentional” and “serious circumstances”. As the judgment of “intentional” is primarily based on indirect and external evidence, and the evidence proving serious circumstances may also reflect the subjective intention of the infringer, the severity of circumstances becomes the primary factor in deciding whether punitive damages apply to intellectual property infringement [5]. Although the judicial interpretation of the Supreme People’s Court lists serious circumstances by way of examples, this “listing method” cannot cover all the serious circumstances of intellectual property infringement. Moreover, fixed examples make it difficult to adapt to the constantly changing judicial practice resulting from social and technological progress [28]. Chinese scholars’ research on identifying serious circumstances in intellectual property punitive damages generally adopts the method of legal hermeneutics, but their interpretative approaches differ. Some scholars begin with legal provisions; for instance, one scholar proposed interpreting serious circumstances according to the three components of liability for intellectual property infringement damages (“infringement of others’ rights”, “damage”, and “causality”) [29]. Others approach punitive damages from a functionalist perspective, suggesting that identifying serious circumstances should align with the objectives of compensation, punishment, and prevention in the punitive damages system for intellectual property infringement [5].Additionally, some scholars adopt a divergent thinking approach, listing the elements involved in identifying serious circumstances. For example, one scholar emphasized that “the court must comprehensively consider all case factors, such as the duration of the infringement, the covered territory, the behavior, and the consequences” to determine whether the intellectual property infringement is “serious” [17].In summary, existing research in China mainly relies on scholars’ subjective inferences, and the identification of serious circumstances lacks a defined decision-making framework.

Based on the review of *BMW of North America, Inc. v. Gore* [30], the United States Supreme Court proposed that the calculation of punitive damages should consider three criteria: (1) the degree or culpability of the defendant’s misconduct; (2) the gap between the damages (or potential damages) suffered by the plaintiff and the punitive damages; and (3) the disparity between the punitive damages awarded by the jury and the civil penalties imposed in similar cases [31]. The Canadian Supreme Court emphasized maintaining rationality in awarding punitive damages through the proportionality principle [32].

China’s research on the compensation amount for punitive damages in intellectual property infringement primarily focuses on the calculation of the base amount and multiplier in judicial practice. Chinese scholars consider the compensation base as the actual loss suffered by the right holder, with the benefit gained by the infringer due to the infringement or the licensing fee multiplier used as a presumption of the actual loss [33,34]. Regarding the compensation multiplier, some scholars propose setting a corresponding punishment multiplier based on the severity of the objective infringement circumstances [35]. Other scholars suggest applying the proportion principle to determine the amount of punitive damages based on the subjective fault and severity of the infringement [36]. Therefore, assessing the severity of the infringement is crucial for adjudicating the amount of punitive damages.

In summary, the legitimacy of applying the punitive damages system for intellectual property infringement is generally recognized, but consensus on the conditions for its application remains unresolved. The punitive damages system serves a strong punitive function. To prevent the abuse and generalization of the punitive damages system within the private law field of intellectual property, it is essential to establish a more objective standard for its application [37]; the presence of serious circumstances is an important factor to this end.

The presence of serious circumstances is a critical factor in determining whether punitive damages can be applied to intellectual property infringement, and the severity of the circumstances plays a key role in deciding the multiplier for punitive damages. However, there is no decision model derived from judicial practice, either internationally or within China, for identifying serious circumstances in intellectual property infringement cases [28,38]. This study aims to address this gap in the research.

Using grounded theory and case statistics methods, this study examined the key elements defining the “seriousness” of intellectual property infringement and the theoretical reasoning behind them, based on judgments involving the application of the punitive damages system in China. The aim was to present a Chinese version of a decision model for determining the “seriousness” of intellectual property infringement that may prove useful worldwide.

### Research design

#### Research methodology.

This study adopts grounded theory method and descriptive statistical analysis to examine judgments in intellectual property infringement cases in China where punitive damages are applied. (1) In Study 1, grounded theory is used to develop a model of “serious circumstances” in intellectual property infringement. Grounded theory, recognized as one of the most scientific qualitative research methods, aims to construct a model from empirical data. The researcher employs an inductive approach to analyze the data without preconceived theories, extracting a related model [39]. One of the prerequisites for applying punitive damages in intellectual property infringement cases is the existence of serious circumstances. However, there is currently no theoretical framework that legally defines what constitutes such circumstances. Through grounded theory, a model of serious circumstances can be derived from prior cases, offering judges and parties a relatively consistent reference for identifying serious circumstances in intellectual property infringement cases. (2) In Study 2, descriptive statistical analysis is used to investigate which serious factors judges prioritize in different types of intellectual property cases and the correlation between these factors and the determination of the punitive damages multiplier.

As the punitive damages system for intellectual property was fully established in China only after the Civil Code’s promulgation in 2020, there is a limited number of cases involving intellectual property infringement with punitive damages. Given the small sample size, regression analysis would yield an insufficient sample and a large margin of error. Therefore, this study adopts the frequency statistics method to observe the situations that Chinese judges tend to rely on when determining the severity of the circumstances in different types of intellectual property infringement cases.

#### Text data collection.

The text data for this study was sourced from the IPHouse database, a Chinese database focused on intellectual property rights [40]. This database includes intellectual property judgment documents as well as search information on patents, trademarks, and geographical indications, etc. It is currently the most mature, professional and comprehensive intellectual property information retrieval system in China. The database contains various intellectual property cases released by courts at all levels in China. The implementation of China’s punitive damages regime for intellectual property rights was fully established after the promulgation of the Civil Code in 2020. Therefore, this research analyzes cases recorded in the IPHouse database from 2021 to 2024. By inputting the keyword “punitive damages” in the “Judgment Results” column and selecting the years 2021–2024 in the “Judgment Year” column, a total of 599 intellectual property case judgments mentioning the term “punitive damages” in the judgment results from 2021 to 2024 were retrieved as of March 9, 2025. After screening each case individually, 245 judgments were identified as involving the application of punitive damages [41]. These included 131 copyright cases, 13 patent cases, 91 trademark cases, 6 trade secret cases, and 4 cases falling outside the above categories.

### Study 1: A grounded theory study on the constituent elements of “serious circumstances” for applying punitive damages in intellectual property infringement cases

In Study 1, approximately two-thirds of the case judgments (160 case judgments) were randomly selected for coding analysis, while the remaining cases (85 case judgments) were reserved for saturation testing.

### Category refinement and model construction

#### Open coding.

During the open coding phase, all selected texts were coded word by word, sentence by sentence, and case by case. This process led to the development of initial concepts and the refinement of categories [42]. For instance, the initial concept of “Online Sales” was derived from the original statement, “Sold online through several e-commerce platforms and numerous stores”, and the initial concept of “Conduct business across provinces” was extracted from the original phrase, “Defendant has many franchisees and a sales network in Liaoning Province, Heilongjiang Province, Xinjiang Province, and Beijing City”. These initial concepts,“Online Sales” and “Conduct business across provinces,” were further grouped into the category of “Infringement space.” This study analyzed the 160 judgments word by word, removed duplicate nodes, and extracted a total of 76 original statements, 49 initial concepts, and 16 initial categories (see Table 1).

**Table 1:** Examples of the category of serious circumstances of punitive damages for intellectual property infringements (partial).

Initial category	Initial concepts	Original statements in textual material
F1 Refuse to cease infringement	f 11 Seizure by public security organisations but refusal to stop infringing	s 11 In October 2018, the defendant was seized by public security authorities. Despite the infringement being investigated and punished, Yang and Hao failed to mitigate the adverse impact in a timely manner and continued to organize large-scale production and sales of counterfeit goods.
	f 12 Refusal to cease infringement when a legal decision or judgment has entered into force	s 12 The defendant refused to comply with a legal ruling and continued to engage in activities prohibited by the injunction.
	...	
F2 Repeated infringement	f 21 Repeated infringement after criminal sanction	s 21 Intentionally infringing on the trademark rights of others on multiple occasions and continuing the infringement even after being subjected to several penalties.
	f 22 Repeated infringement after civil penalty	s 22 Committed in the mediation agreement not to infringe upon plaintiff’s unique packaging and decoration of well-known products, but still used them without authorisation after compensation was paid.
	...	
F3 Obstruction of proof	f 31 Refusal to submit evidence	s 31 The defendant refused to provide relevant accounting books and original documents during the trial stage without any valid reason.
	f 32 Falsification of infringement evidence	s 32 The defendant’s vanillin production line involved numerous non-standard pieces of equipment and several implausible claims. The defendant’s assertion that vanillin-related production equipment could be quickly manufactured without equipment diagrams is clearly unreasonable.
	...	
F4 Dishonest behaviours	f 41 Deliberate delay of proceedings	s 41 The defendant maliciously filed a request to invalidate the patent, with the clear intention of delaying the litigation, demonstrating obvious malice from Guangdong and Anhui Meibo Company.
	f 42 Non-fulfilment of commitments	s 42 Kepai Company’s failure to honor a settlement agreement and its disregard for the intellectual property rights of others violates the principle of good faith, and the malicious intent behind the infringement is highly serious.
	...	
F5 The Relationship between the defendant and the plaintiff	f 51 There was once a licensing relationship between the defendant and the plaintiff	s 51 The defendant had previously been a franchisee of the plaintiff, with the right to use the registered trademark involved in the case. After the termination of the franchise agreement, the defendant continued to use the plaintiff’s trademark without consent.
	f 52 There was once a labor relationship between the defendant and the plaintiff	s 52 Zhang Yong had previously been employed by Aichuang Company, serving as the regional general manager and the general manager of the sales and service center. During his tenure, he had access to the software involved in the case.
	...	
F6 The popularity of the infringed intellectual property	f 61 The reputation of the infringed intellectual property has spread widely	s 61 Several of the plaintiff’s works have been extensively reported by national and provincial media outlets.
	f 62 The reputation of the infringed intellectual property has a long history	s 62 The plaintiff is a state-controlled, publicly listed company with a rich 400-year history in winemaking. It has consistently produced high-quality wines, earning numerous world-class and national awards, and is highly regarded in the industry.
	...	
F7 Take infringement as a profession	f 71 Infringing goods account for the major proportion	s 71 Infringing goods make up a significant portion of the defendant’s main business.
	f 72 The company’s profits mainly come from infringement	s 72 The defendant, Zhenjiang Schneider Company, derives the majority of its profits from infringement, which constitutes a professional practice of infringement.
	...	
F8 Infringement space	f 81 Online Sales	s 81 The defendant sold goods through multiple e-commerce platforms and numerous online stores.
	f 82 Conduct business across provinces	s 82 The defendant has numerous franchisees and a sales network across Liaoning Province, Heilongjiang Province, Xinjiang Province, and Beijing City.
	...	
F9 Infringement time	f 91 Duration of infringement	s 91 The infringing activities of Jingbaidu Company and its branch offices had not been fully discontinued by the time of the first-instance litigation. As a result, the period of infringement in this case lasted approximately 63 months, equivalent to 5.25 years.
	f 92 Frequency of infringement	s 92 The defendant, Ma Qingsheng, has repeatedly engaged in trademark infringement on numerous occasions over a period exceeding two years.
F10 Infringement scale	f 101 The business premises of the infringer	s 101 The infringing activities in this case took place in a commercial center, with a registered business area exceeding 2,000 square meters.
	f 102 Number of infringing stores	s 102 The infringer operated several stores involved in the alleged infringement.
	....	
F11 The range of people affected by infringement	f 111 Infringement affects franchisees	s 111 Infringement affects both end consumers and intermediate franchisees.
	f 112 The infringement affects enterprises in the middle and downstream of the industrial chain	s 112 The defendant company, as a manufacturer of the infringing goods, serves as the source of the infringement.
	...	
F12 The manner of infringement	f 121 Diversity of the infringement act	s 121 The infringement involved not only using the trademark on identical or similar goods, but also registering the trademark as a domain name and enterprise name.
	f 122 Infringing private rights while violating administrative regulations	s 122 Counterfeiting, altering, buying, selling, or renting seed production and management licenses constitutes an aggravating circumstance in the infringement.
	...	
F13 Damage to the right holder	f 131 The loss of business opportunities	s 131 Within less than two months of its establishment, the defendant quickly capitalized on business opportunities worth nearly 20 million yuan that originally belonged to the plaintiff, causing substantial economic losses to the plaintiff.
	f 132 Damage to business reputation	s 132 The defendant manufactured and sold counterfeit well-known liquor, offering goods similar to those of the trademark owner, but of significantly lower quality. These actions seriously damaged the market reputation of the plaintiff and the Maotai brand, making the infringement extremely severe.
	...	
F14 Infringers’ profits	f 141 Infringement sales amount	s 141 The quantity of infringing products sold reached at least 180,000 units, with sales totaling no less than 620 million yuan.
	f 142 Profits from infringement	s 142 The total profit generated from the infringement amounted to $12 million.
	...	
F15 Disturbing the public order	f 151 Disturbing the market order	s 151 The infringer produced and operated seeds without acquiring the necessary seed production and operation license, and sold the seeds in unmarked packaging.
	f 152 Disrupting the industry’s healthy development	s 152 The defendant engaged in trademark infringement and unfair competition by forging numerous documents, deceiving the platform into passing the audit, and using the plaintiff’s corporate name, registered trademark, and the name of a highly popular game. This not only infringed on the plaintiff’s rights and misled consumers but also openly challenged the law, severely violated business ethics and good faith principles, and undermined the competitive order in the online environment, as well as the social integrity system.
	...	
F16 Damage to the public interests	f 161 Endangering human health	s 161 The product involved in the infringement is an ultrasonic therapy instrument, which is used directly on the human face in the medical beauty industry. As such, this infringement poses a potential risk to human health.
	f 162 Harming consumer interests	s 162 The infringer provides alcoholic products of inferior quality, leading to consumer losses. Some consumers have recovered part of their losses through refunds and returns.
	...	

#### Axial coding.

Axial coding was employed to establish “a dense network of relationships around the ‘axis’ of the category” [43]. In this stage, the coding from the previous phase was further analyzed and summarized [42]. For instance, the infringer “refuses to stop infringing” after being discovered or sued, “repeats the infringement” despite being penalized, “obstructs the proof” during litigation, or engages in “dishonest behaviour”, such as disregarding the commitment and settlement agreement, these actions reflect the infringer’s serious “subjective malevolence” based on the objective facts. Therefore, punitive damages should be imposed. Following this analysis and synthesis, the initial categories were consolidated into three main categories: subjective malevolence, degree of behavior, and resulting damage (see Table 2).

**Table 2:** Main categories for the spindle code formation.

Main category	Corresponding category	Scope connotation
Z1 Subjective malevolence	F1 Refusal to cease infringement	Refusal to cease infringement refers to the infringer’s continued infringement after being discovered or prosecuted, or despite the enforcement of a mediation agreement, legal ruling, or judgment.
	F2 Repeated infringement	Repeated infringement occurs when the infringer, after facing criminal, civil, or administrative penalties, fails to repent and continues committing the same infringement.
	F3 Obstruction of proof	Obstruction of proof involves actions such as falsifying, destroying, concealing, or refusing to submit evidence of infringement, which may result in damages being unquantifiable.
	F4 Dishonest behaviours	Dishonest behavior refers to violating the fundamental principle of honesty and trust, treating national laws or issued commitments and settlement agreements with disregard, reflecting serious subjective malice.
	F5 The relationship between the defendant and the plaintiff	When there exists or existed a labor, service, cooperation, licensing, distribution, agency, representative, or other relationships between the defendant and the plaintiff or an interested party, and the defendant had access to the infringed intellectual property rights.
	F6 The popularity of the infringed intellectual property object	The infringed intellectual property object is relatively well-known. The defendant should have been aware that it belonged to another party’s intellectual property rights but chose to infringe, which reflects the presence of subjective intent.
Z2 Degree of conduct	F7 Take infringement as a Profession	The infringing goods make up a significant portion of the defendant’s main business, or the defendant’s profits primarily stem from the infringing act, which constitutes taking infringement as a profession.
	F8 Infringement space	The spatial scope of infringement encompasses both physical and virtual (cyber) aspects.
	F9 Infringement time	The time range of infringement includes both the duration and frequency of the infringement.
	F10 Infringement scale	The scale of infringement includes the size of the infringer’s production and operation sites, the number of stores, as well as the types and quantities of infringing goods manufactured and sold.
	F11 The range of people affected by infringement	The infringement not only affects competitors and rights holders but also consumers, distributors, and franchisees within the industry chain. The closer the infringer is to the upstream of the industry chain, the more distributors or franchisees involved, thereby widening the scope of the infringement’s impact.
	F12 The manner of infringement	The manner of infringement refers to the methods by which the infringement is committed or the acts involved. Infringement involves unauthorized copying, use, sale, and other exploitation of intellectual property. The means of infringement refer to improper methods or measures used to carry out the infringement.
Z3 Resulting damage	F13 Damage to the right holder	The damage to the right holder can be considered from two aspects: one is the loss of vested or potential economic benefits, goodwill, or market leadership; the other is the loss arising from the prior investment in the right holder’s intellectual property rights, market goodwill, accumulated market leadership, and the costs incurred in defending their rights.
	F14 The infringer’s gains	The infringer’s gains can be assessed in terms of the infringer’s sales amount, sales volume, profit earned, and other related factors.
	F15 Disturbing public order	Disturbing public order includes disrupting the market order, the order of industrial development, and industry regulations, among other things.
	F16 Damage to the public interests	The infringer’s actions not only harm the interests of the right holder but may also jeopardize public interests. For example, substandard products threaten public health and safety, while counterfeit products mislead consumers into purchasing inferior goods.

#### Selective coding and model building.

**Fig 1:**
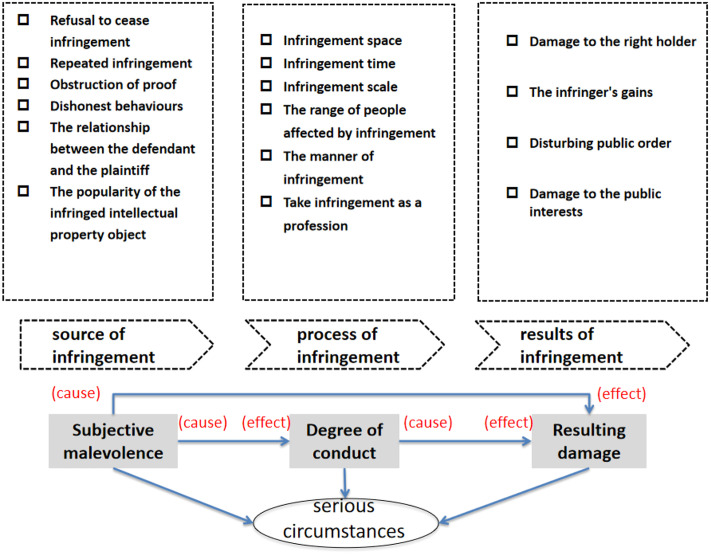
Key elements of the serious circumstances determinations of punitive damages for intellectual property infringement based on grounded theory.

Selective coding refers to the process of identifying a core category after systematically analyzing all the discovered categories and examining the inner connections between the core category and the fragmented concepts, forming a “story line” [42]. This study takes the determination of serious circumstances for the application of punitive damages in intellectual property infringement cases as the core category. The “story line” surrounding the core category is structured as follows(see Fig 1): “subjective malevolence”, “degree of conduct”, and “resulting damage” determine the serious circumstances based on the source, the process, and outcome of the infringement, respectively. The three key elements of “subjective malevolence”, “degree of conduct”, and “resulting damage” are interrelated and exhibit reciprocal causal relationships. In practice, judges typically assess whether a case involves serious circumstances as a whole by examining each sub-element of “subjective malevolence”, “degree of conduct”, and “resulting damage”. By studying the key elements of serious circumstances, the theoretical logic behind each, and the sub-elements, the accuracy and objectivity of the determination of serious circumstances can be enhanced. This approach helps prevent the abuse and overgeneralization of the punitive damages system in the field of intellectual property rights.

### Theoretical saturation test

Theory saturation refers to the continuous comparison of conceptual indicators in textual data until these indicators no longer lead to further theoretical specification or refinement [44]. To test the theoretical saturation of the model for determining the “seriousness” of intellectual property infringement, this study analyzed 85 reserved cases using open, axial, and selective coding methods as described above. We did not identify new concepts or categories, nor did we find new relationships between the categories. Additionally, two field experts affirmed the coding results and the model. Therefore, the “seriousness” judgment model for intellectual property infringement successfully passed the theoretical saturation test.

### Model interpretation

The objective requirement for applying punitive damages in intellectual property infringement cases is “seriousness”. Whether and to what degree “circumstances are serious” is determined by a comprehensive consideration of multiple factors. These evaluation elements are interrelated and interact with one another. In judicial practice, the law applicator must determine if the intellectual property infringement meets the threshold for serious circumstances based on dynamic systems theory [28]. Only through a holistic and comprehensive judgment can the rationality of applying the punitive damages system in intellectual property infringement cases be ensured, as well as the accuracy and rigor of calculating the base and multiplier of damages. A grounded theory analysis of Chinese judgments revealed that the “seriousness” judgment model for punitive damages in intellectual property infringement consists of three dimensions: subjective malevolence, degree of behaviour, and resulting damage.

### Subjective malevolence

The legitimacy of the punitive damages system is grounded in the culpability of subjective malevolence. Punishing the culpable is a key cornerstone in the establishment of punitive damages for intellectual property infringement. Punitive damages are designed not only to cover actual losses but also to strongly condemn the infringer for intentionally violating intellectual property rights without regard for the consequences. Society permits punishment as a means to prevent greater harm. The infringer’s deep subjective malice is the root cause of repeated and incorrigible infringement: “Several times intentionally committing infringement of the trademark rights of others and continuing to commit infringement even after being sentenced to several penalties” (s 21).

Foreign intellectual property legislation typically requires “maliciousness” for the application of punitive damages. For example, Article 97(2) of the United Kingdom’s Copyright, Designs, and Patents Act 1988 stipulates that one of the factors the court must consider when awarding additional damages is “the flagrancy of the infringement”. In American copyright practice, judges also regard maliciousness as a prerequisite for punitive damages liability [45]. Punitive damages should only be imposed on infringers whose actions are driven by relatively large subjective malevolence, resulting in severe consequences [46]. Limiting the application of punitive damages to malicious infringement is more conducive to realizing their social control function.

Through grounded theory analysis of judgments in China, the subjective malevolence of infringers is primarily reflected in their refusal to cease infringement, repeated infringement, obstruction of proof, dishonest behavior, and other objective factors. Refusal to cease infringement occurs when infringers continue infringing even after the infringements have been discovered, prosecuted, or even after mediation agreement, legal rulings, or judgments have come into effect. Repeated infringement refers to situations where infringers, after facing criminal, civil, or administrative penalties, persist in committing the same infringement without repentance. Obstruction of proof involves actions such as forging, destroying, or concealing evidence, or refusing to submit evidence of the infringement, leading to a situation where it becomes impossible to assess the infringement losses. Dishonest behaviour contradicts the fundamental principles of honesty and trust. It refers to actions that disregard national laws, effective commitments, or settlement agreements, and are driven by serious subjective malice. Furthermore, when there exists or has existed a relationship between the defendant and the plaintiff or an interested party (such as labor, service, cooperation, licensing, distribution, agency, or representation), and the defendant had access to the infringed intellectual property rights, or when the plaintiff’s intellectual property is relatively well-known, it can be inferred that the defendant was aware that using the plaintiff’s intellectual property constituted an infringement but chose to proceed with it. This reflects the defendant’s subjective malevolence, as it shows intentional infringement despite knowing the rights involved.

There is a certain causal mapping relationship between “subjective malevolence” and “degree of behaviour” and “resulting damage”. Generally, the deeper the subjective malevolence, the more serious and egregious the infringement, and the greater the resulting damage. However, this causal mapping relationship is only a general correlation and may not apply in all cases. The amount of punitive damages is often positively correlated with the degree of culpability, the degree of behaviour and resulting damage.

The term “malicious intent” used here to determine the “seriousness of circumstances” differs from the concept of “wilful intent” in the Chinese Civil Code, Patent Law (revised in 2020), and the Copyright Law (revised in 2020).“Willful intent” refers to the clear awareness of a legal prohibition, with the infringer intentionally violating it. While “wilful intent” focuses on the infringer’s cognition, “malicious intent” reflects the infringer’s malicious motives in committing the act. This represents a deeper degree of subjective intent, supported by objective external evidence. For instance, the “repeated infringement” situation reflects the intentional infringement motive from an objective perspective, but in essence, it is a specific manifestation of serious circumstances. Demonstrating the presence of subjective “maliciousness” through external serious circumstances helps to mitigate the uncertainty associated with applying subjective requirements.

### Degree of conduct

The degree of the infringer’s behaviour determines the serious circumstances of intellectual property infringement from a procedural perspective, facilitating both quantitative and qualitative analysis. The degree of behavior is primarily reflected in factors such as the spatial scope, the time frame, the scale of infringement, the range of people affected, the mode of infringement, and whether the infringement is pursued as a profession. The spatial scope of infringement includes both physical and virtual (network) space. The time frame of infringement encompasses both the duration and frequency of the infringement. The scale of infringement involves the area of the infringer’s production and operation sites, the number of stores, and the type and quantity of infringing goods manufactured and sold. The range of people affected by the infringement refers to the fact that the infringement impacts not only competitors and right holders, but also consumers, distributors, franchisees, and others in the industrial chain. The mode of infringement refers to the acts or methods employed by the infringer to carry out the infringement. Taking infringement as a profession means that infringing goods constitute a significant portion of the infringer’s main business, or the defendant’s profits primarily derive from the infringing act. In general, the longer and more frequent the infringement, the broader the infringement scope (e.g., not only physical space but also cyberspace), the larger the scale of infringement, the wider the range of people affected by the infringement(the closer the infringer is to the upstream of the industry chain, the more distributors and franchisees involved), the more diverse the infringement methods, and the more the infringer treats infringement as a profession, the more serious the circumstances. For example, “not only using the trademark on the same or similar goods, but also using the trademark by registering a domain name, enterprise name, etc., …” (s 121). Such a variety of infringements would be closer to serious circumstances than merely “using a trademark on similar goods”.

The basic attributes and forms of the infringement and the circumstances in which the infringer uses the intellectual property, determine the apparent degree of infringement, which further influences the extent of damage to the right holder and the potential and degree of impact on public order and interests [47]. There is also a certain causal relationship between the “degree of conduct” and the “results of the damage”. Generally, the more serious and egregious the acts of infringement, the greater the resulting damage. However, this causal relationship is not always established in specific cases. If the infringer can prove that their actions did not cause serious damage to the rights holder in a particular case, the judgment of the severity of the circumstances should be adjusted accordingly [28].

“Degree of conduct” is a key element in determining the “seriousness” from a procedural perspective, as it can restore and visualize the tort process, making the degree of the tort quantifiable. The application of punitive damages in intellectual property infringement primarily relies on the fact that punitive damages can serve as a deterrent to prevent and discourage the occurrence of infringement. However, intellectual property law is part of private law. Infringement compensation within private law is based on compensatory damages, with punitive damages serving as a supplementary measure. Even in common law countries where punitive damages are widely applied across all areas of private law, judges remain cautious in their use of punitive damages [38]. Judge Devlin of the Supreme Court of the United Kingdom believed that “the power to award exemplary damages is a weapon that can be used both to preserve freedom and to combat it” [48]. Although punitive damages can promote social justice, their abuse may lead to unjust outcomes. Therefore, judges should exercise caution and restraint determining the multiplier for punitive damages. Considering the “degree of conduct” as one of the factors to determine the “seriousness” of infringement can make the application of punitive damages more reasonable, moderate, and operable.

### Resulting damage

The resulting damage is used to determine the “seriousness” of intellectual property infringement from the dimension of the results. In assessing the “seriousness”, damage is the most direct element and holds the most significant position. The factors for consideration include “damage to the right holder”, “The infringer’s gains”, “the disturbance of public order”, and “damage to public interests”.

The damage to the right holder can be examined from two aspects. On one hand, it can be considered in terms of explicit economic losses suffered by the right holder. On the other hand, it can be assessed in terms of hidden losses, such as the right holder’s prior investment in intellectual property, accumulated market goodwill, the market leadership. The infringer’s gains can be considered in terms of factors such as the infringer’s sales amount, sales quantity, transaction amount, transaction volume, business volume, profit, and so on. The disturbance of public order encompasses disruptions to market order, industrial development, and industry rules. The damage to public interests refers to the fact that the infringement not only harms the rights of the obligee but may also damage public interests. For instance, substandard products jeopardize public health and safety, while counterfeit products mislead consumers. In general, the greater the damage to the obligee, or the more significant the infringer’s gains, the greater the damage to public interests, or the more severe the disturbance of public order, the more serious the infringement.

There was a significant positive correlation between the damage outcome and the degree of conduct (such as the duration, scope, and scale of the tort). However, there were also cases where the tort was not prolonged, widespread, or large in scale, yet it still resulted in severe damage. Therefore, the damage outcome must be considered independently when determining serious circumstances [5].

The damage result encompasses both the explicit and implicit losses suffered by the right holder. The explicit loss serves as the basis for calculating punitive damages. Punitive damages have the purpose and function of effectively compensating the right holder and curbing the recurrence of infringement. On one hand, due to the intangible and abstract nature of intellectual property rights, identifying and proving the right holder’s loss in such cases can be difficult. Implicit losses include damage to goodwill, loss of market dominance, and the forfeiture of potential trading or licensing opportunities. Therefore, when the right holder’s loss is significant or the infringer’s profit is substantial (which indicates the right holder’s serious losses from another perspective), it is necessary to assess the right holder’s loss using a multiplier of the actual loss to ensure full compensation. On the other hand, punitive damages raise the cost of the infringer’s violation, causing the infringer to cease similar infringing activities due to the lack of economic incentive. Furthermore, punitive damages serve as a warning and deterrent, encouraging potential infringers to abandon their intentions and actions. Thus, punitive damages can effectively contain and prevent infringement [5]. Posner once noted that in cases where the cost of infringement is very low, it is essential for the court to apply punitive damages to maximise efficiency through voluntary market transactions [49].

The damage results include not only the losses suffered by the right holder but also those incurred by society, such as the adverse effects on the industry and even society at large. As a form of social damage compensation [50], when the infringement jeopardizes public health, endangers public safety, or seriously disrupts the industry or market order, the judge may impose punitive damages on the infringer to educate and guide the public, purify the market environment, and cultivate a culture of respect for intellectual property rights [50]. For example, in the trademark infringement case [51] between *Alcera* and *Guangzhou Kepai Industry Co., Ltd.*, the infringing goods were ultrasound therapeutic devices used in the medical beauty industry, directly applied to the human face. The court ruled that labeling counterfeit ultrasound devices as genuine could pose a risk to human health. In the trademark infringement case [52] between *Opple Lighting Co. Ltd.* and *Guangzhou Huasheng Plastic Products Co., Ltd.*, the court determined that substandard lamp products could lead to safety accidents, threaten public safety, and result in serious consequences. In the case of *Jiangsu Jindi Seed Industry Technology Co., Ltd.* v *Jiangsu Qinggengtian Agricultural Industry Development Co., Ltd*. regarding the infringement of the right to new plant varieties, [53]the court held that the defendant’s unauthorized production and sale of seeds would disrupt market order. The social impact demonstrated in these three cases was considered by the judges as key factors in determining serious circumstances and applying punitive damages.

### Study 2: A descriptive statistical analysis of the key elements constituting “serious circumstances” in various types of intellectual property infringement cases subject to punitive damages

#### Percentage statistics.

**Fig 2:**
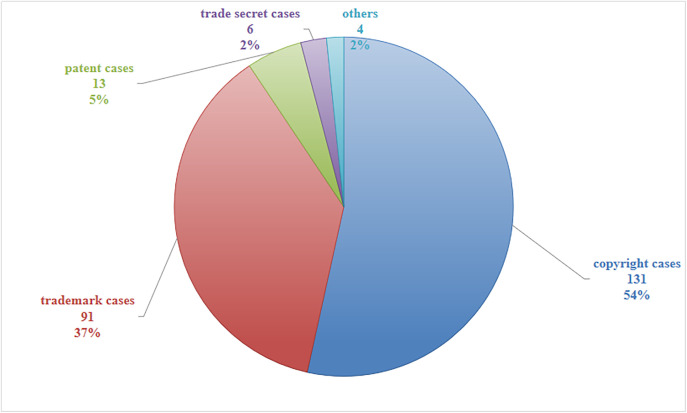
Proportion of different types of cases.

Study 2 continues with a descriptive statistical analysis of the 245 case judgments from Study 1, as mentioned above. The analysis revealed that among intellectual property cases involving punitive damages, copyright cases were the most numerous (131 cases, accounting for 54%), followed by trademark cases (91 cases, accounting for 37%), patent cases (13 cases, 5%), and trade secret cases (6 cases, 2%).(see Fig 2)

#### Frequency and proportion statistics.

Based on Study 1, Study2 analyzes the frequency and proportion of serious elements considered by judges when applying punitive damages. First, frequency and proportion statistic are conducted without distinguishing between compensation multiples, followed by statistics that differentiate between single-fold compensation and multi-fold compensation. Finally, Table 1 and Table 2 are generated. Table 1 shows the serious circumstances that led judges to apply punitive damages in different types of intellectual property cases, while Table 2 shows the circumstances that led judges to increase the punitive damages multiple in different types of intellectual property cases. Regarding the numbers in the tables, for example, in Table 1, the number 111 indicates that, among the 131 copyright cases where punitive damages were applied, the “Refusal to cease infringement”(F1) occurred in 111 cases, which was a factor for judges to apply punitive damages, and these 111 cases accounted for 85% of the 131 cases.

### Frequency and proportion analysis without differentiation by damages multiples

In copyright infringement cases, judges primarily apply punitive damages based on the infringer’s subjective malice, particularly the “refusal to stop infringement” and “repeated infringement”, both of which reflect the infringer’s wrongful intent. Among the 131 copyright infringement cases involving punitive damages, judges considered the infringer’s “refusal to stop the infringement” as one of the factors in applying punitive damages in 111 cases (85% of the total). In 115 cases (88% of the total), judges considered the infringer’s “repeated infringement” as a factor in applying punitive damages.

In trademark infringement cases, the factors considered by judges when applying punitive damages are relatively scattered. However, it can still be observed that in over 40% of cases, judges consider factors such as the “refusal to cease infringement”, “the popularity of the infringed object”, as well as “the scope of infringement”, “the duration of infringement” and “the scale of infringement” when applying punitive damages.

In patent infringement cases, judges primarily apply punitive damages based on the infringer’s subjective malice, particularly the “refusal to cease infringement”, as well as the degree of the “duration of infringement” and “scale of infringement”. In over 50% of the cases, judges consider these three factors, among others, when applying punitive damages.

In trade secrets cases, judges almost always cite “the infringer’s dishonest behavior”, “the existence of a labor, cooperation, agency, or other relationship between the plaintiff and the defendant”, and “the significant damage caused to the right holder” as primary reasons for applying punitive damages, along with other factors.

### Frequency and proportion analysis categorized by single-fold compensation (1×) and multi-fold compensation (>1×)

Based on the above statistics, as the multiple of punitive damages for copyright infringement increases, the judges primarily focus on the following factors: “F3 Obstruction of proof,” “F5 The relationship between the defendant and the plaintiff,” and “F6 The popularity of the infringed intellectual property” under the category of “Z1 Subjective malevolence”; “F7 Infringement space,” “F8 Infringement time,” “F9 Infringement scale,” and “F12 Take infringement as a profession” under the category of “Z2 Degree of conduct”; and “F15 Disturbing public order” under the category of “Z3 Resulting damage”.

In trademark infringement cases, as the multiple of punitive damages increases, judges primarily focus on the following factors: “F10 The range of people affected by infringement” and “F11 The manner of infringement” under the category of “Z2 Degree of conduct.” Additionally, it is evident that when the duration and scale of infringement double, the multiple of punitive damages also doubles accordingly.

In patent infringement cases, as the multiple of punitive damages increases, judges primarily focus on the following factors: “F1 Refusal to cease infringement” under the category “Z1 Subjective malevolence,” and “F8 Infringement time” and “F9 Infringement scale” under the category of “Z2 Degree of conduct”.

In cases of multiple punitive damages for trade secret infringement, judges pay as much attention to the elements “F4 Dishonest behaviors,” “F5 The relationship between the defendant and the plaintiff,” and “F13 Damage to the right holder” as they do in cases involving single damages. Additionally, judges also place emphasis on the elements “F8 Infringement time” and “F9 Infringement scale” under the category of “Z2 Degree of conduct.”

“Serious Circumstances” impact the multiplier of punitive damages, thereby influencing the total amount. While there is a correlation between “Serious Circumstances” and the compensation multiplier, it is difficult to express the relationship between the two through a definitive function in judicial practice. It is not appropriate to simply apply some formulas based on the relationship between punitive damages and damages multipliers [54]. The determination of the specific multiple for punitive damages primarily depends on the judge’s assessment of whether the purpose of punitive damages has been achieved. In other words, the judge should connect the case facts with the objective of punitive damages and evaluate how the ruling on punitive damages contributes to realizing this objective [54]. The Canadian Federal Supreme Court applied the principle of proportionality in the *Whiten* case, stating that punitive damages should be proportional to the culpability of malicious unlawful acts and reasonably aligned with the goals of punishment and deterrence.

The statistical results from Table 1 and Table 2 show that when judges apply punitive damages in intellectual property infringement cases, they do not consider a single factor but instead take multiple factors into account comprehensively. Furtherover, in different types of intellectual property cases, the weight of each severity factor in the application of punitive damages by judges varies.

### Discussion on differences across intellectual property types

The elements for determining “serious circumstances” in intellectual property infringement cases are diverse. For different types of cases, when judges apply punitive damages, the weight of various elements in the “serious circumstances” evaluation system differs, with those elements that align with the purpose of the punitive damages system carrying greater weight.

Punitive damages serve three primary purposes: deterrence, compensation, and protection of public interest. (1) Deterrence: by requiring malicious infringers to pay amounts exceeding actual losses, the system curbs intentional and repeat infringement. Raising the cost of infringement deters speculative conduct in cases where “the profits from infringement exceed the damages awarded.” (2) Compensation: beyond compensating the right holder for actual losses (such as reduced profits and rights-protection costs), additional compensation is also provided to redress hard-to- quantify hidden harms, including damage to business reputation and lost market opportunities. Compensating for hidden damage through punitive damages can enhance right holders’ confidence in the intellectual property protection system and thereby encourage investment in innovation. For example, high-tech firms can better recover research and development expenditures through enhanced awards. (3) Safeguarding the public interest: by targeting unauthorized use and counterfeiting, the punitive damages system promotes market order and a fair competitive environment, sending a clear signal that “intellectual property must be protected and innovation respected.”

Across different areas of intellectual property, the objectives of punitive damages differ because the nature of the rights, the patterns of infringement, and their social impact vary. Accordingly, when judges assess whether infringement amounts to “serious circumstances,” the factors they emphasize also differ. Conversely, by analyzing the weights that judges assign to each element in the “serious circumstances” evaluation systems across different types of IP cases (Tables 3 and 4), one can infer the ordering of punitive-damages objectives across case types (Table 5).

**Table 3:** The frequency and proportion of serious circumstances considered by judges when applying punitive damages.

Main category	Corresponding category	copyright cases		trademark cases		patent cases		trade secret cases	
		Number	Proportion	Number	Proportion	Number	Proportion	Number	Proportion
Z1 Subjective malevolence	F1 Refusal to cease infringement	**111**	**85%**	**44**	**48%**	**12**	**92%**	1	17%
	F2 Repeated infringement	**115**	**88%**	14	15%	6	46%	1	17%
	F3 Obstruction of proof	2	2%	12	13%	1	8%	1	17%
	F4 Dishonest behaviours	0	0%	3	3%	2	15%	**6**	**100%**
	F5 The relationship between the defendant and the plaintiff	7	5%	22	24%	5	38%	**6**	**100%**
	F6 The popularity of the infringed intellectual property object	7	5%	**44**	**48%**	1	8%	0	0%
Z2 Degree of conduct	F7 Infringement space	5	4%	**37**	**41%**	5	38%	0	0%
	F8 Infringement time	11	8%	**42**	**46%**	**7**	**54%**	**4**	**67%**
	F9 Infringement scale	8	6%	**38**	**42%**	**9**	**69%**	**3**	**50%**
	F10 The range of people affected by infringement	1	1%	20	22%	3	23%	0	0%
	F11 The manner of infringement	2	2%	18	20%	1	8%	0	0%
	F12 Take infringement as a profession	2	2%	23	25%	0	0%	1	17%
Z3 Resulting damage	F13 Damage to the right holder	0	0%	0	0%	0	0%	**6**	**100%**
	F14 The infringer’s gains	2	2%	17	19%	1	8%	0	0%
	F15 Disturbing public order	3	2%	7	8%	0	0%	1	17%
	F16 Damage to the public interests	0	0%	12	13%	0	0%	0	0%

**Table 4:** The frequency and proportion of serious circumstances considered by the judge when determining the multiple of punitive damages(Number, Proportion).

Main category	The impact factors to be considered when applying punitive damages	copyright cases		trademark cases		patent cases		trade secret cases	
		compensation multiple is ≤ 1 (with a total of 120 cases)	compensation multiple is > 1 (with a total of 11 cases)	compensation multiple is ≤ 1(with a total of 40 cases)	compensation multiple is > 1 (with a total of 51 cases)	compensation multiple is ≤ 1(with a total of 3 cases)	compensation multiple is > 1 (with a total of 10 cases)	compensation multiple is ≤ 1(with a total of 1 cases)	compensation multiple is > 1 (with a total of 5 cases)
Z1 Subjective malevolence	F1 Refusal to cease infringement	**108**, **90%**	3, 27%	14, 35%	**30, 59%**	2, 67%	**10, 100%**	0, 0%	1, 20%
	F2 Repeated infringement	**110, 92%**	5, 45%	6, 15%	8, 16%	1, 33%	5, 50%	0, 0%	1, 20%
	F3 Obstruction of proof	0, 0%	2, 18%	4, 10%	8, 16%	0, 0%	1, 10%	0, 0%	1, 20%
	F4 Dishonest behaviours	0, 0%	0, 0%	1, 3%	2, 4%	1, 33%	1, 10%	**1, 100%**	**5, 100%**
	F5 The relationship between the defendant and the plaintiff	3, 3%	**4, 36%**	9, 23%	13, 25%	0, 0%	5, 50%	**1, 100%**	**5, 100%**
	F6 The popularity of the infringed intellectual property object	3, 3%	**4, 36%**	16, 40%	**28, 55%**	1, 33%	0, 0%	0, 0%	0, 0%
Z2 Degree of conduct	F7 Infringement space	2, 2%	**3, 27%**	16, 40%	21, 41%	1, 33%	4, 40%	0, 0%	0, 0%
	F8 Infringement time	8, 7%	**3, 27%**	17, 43%	25, 49%	0, 0%	**7, 70%**	0, 0%	**4, 80%**
	F9 Infringement scale	4, 3%	**4, 36%**	12, 30%	**26, 51%**	1, 33%	**8, 80%**	0, 0%	**3, 60%**
	F10 The range of people affected by infringement	1, 1%	0, 0%	6, 15%	14, 27%	0, 0%	3, 30%	0, 0%	1, 20%
	F11 The manner of infringement	1, 1%	1, 9%	6, 15%	12, 24%	0, 0%	1, 10%	0, 0%	0, 0%
	F12 Take infringement as a profession	0, 0%	2, 18%	12, 30%	11, 22%	0, 0%	0, 0%	0, 0%	1, 20%
Z3 Resulting damage	F13 Damage to the right holder	0, 0%	0, 0%	0, 0%	0, 0%	0, 0%	0, 0%	**1, 100%**	**5, 100%**
	F14 The infringer’s gains	1, 1%	1, 9%	10, 25%	7, 14%	1, 33%	0, 0%	0, 0%	0, 0%
	F15 Disturbing public order	0, 0%	**3, 27%**	2, 5%	5, 10%	0, 0%	0, 0%	0, 0%	1, 20%
	F16 Damage to the public interests	0, 0%	0, 0%	3, 8%	9, 18%	0, 0%	0, 0%	0, 0%	0, 0%

**Table 5:** Ordering of punitive-damages purposes across IP case types.

Intellectual Property Types	Ordering of purposes
Copyright	deterrence>safeguarding the public interest> compensation
Patent	deterrence>compensation> safeguarding the public interest
Trademark rights	deterrence>compensation≈ safeguarding the public interest
Trade secrets	deterrence>compensation> safeguarding the public interest

As shown in Table 3, across all categories of intellectual property infringement, judges regard “Z1:subjective malevolence” as the most important factor when applying punitive damages. However, both the emphasis placed on this factor and the subfactors used to substantiate subjective malevolence vary by case type. Accordingly, the predominant purpose of punitive damages in IP infringement is to fight against malicious infringement and curb future infringements.

By contrast, judges give far less weight to “Z3: resulting damage” than to “Z1: subjective malevolence.” Within Z3, only the sub-element “F13: damage to the right holder” is treated as a key factor for applying punitive damages in trade-secret infringement, and “F15: disturbing public order” is treated as a relatively key factor in copyright cases where multiple punitive damages are imposed; in patent and trademark infringement, Z3 does not rank among the top factors in the “serious circumstances” evaluation system. Judges’ attention to “F13: damage to the right holder” and “F14: the infringer’s gains” within Z3 reflects the compensatory purpose of punitive damages, while attention to “F15: disturbing public order” and “F16: damage to the public interests” reflects the importance of safeguarding the public interest in punitive damages. Judges’ consideration of “F13: Damage to the right holder” and “F14: The infringer’s gains” within “Z3: Resulting damage” reflects the weight of the compensatory purpose in punitive damages, while their consideration of “F15: Disturbing public order” and “F16: Damage to the public interests” reflects the weight placed on safeguarding the public interest in punitive damages.

“Z2: Degree of conduct” is a procedural element and does not directly indicate the primary purposes of punitive damages. Nonetheless, Z2 is closely tied to the gravity of the infringement. Accordingly, in cases where punitive damages are calculated by applying a multiplier, judges pay particular attention to Z2’s sub-elements, especially F7, F8, and F9, which often influence the multiplier selected.

As shown in Table 5, across all categories of intellectual property infringement, the primary purpose of punitive damages is “deterrence”: the foremost task of modern tort law is to prevent and avert harm. Deterrence can be divided into specific and general forms. Specific deterrence operates by increasing the cost of unlawful conduct, thereby discouraging infringers in individual cases. General deterrence seeks to suppress infringing activity and generate a broader, society-wide deterrent effect of the law, serving as a caution to the public at large [55].

Across different categories of intellectual property, the principal distinction in the purposes of punitive damages lies in the relative priority accorded to “compensation” versus “protection of the public interest.” These differences arise from the distinct attributes of each right. Patent rights falls within industrial property, and trade secret misappropriation is itself a form of unfair competition. In patent and trade secret cases, right holders typically suffer substantial hidden losses; accordingly, judges treat compensation for those hidden losses as the second-ranked purpose for imposing punitive damages. By contrast, trademarks are more closely connected to the general public. Although trademark rights are also industrial property, punitive damages in trademark cases tend to assign roughly equal weight to compensatory aims and to safeguarding the public interest. Unlike industrial property rights, copyright protects works, and the economic value implicated in copyright infringement is generally not substantial. As a result, the compensatory function of punitive damages is less prominent in copyright cases, while deterrent function assumes greater emphasis. Moreover, because copyright also serves to facilitate the proper dissemination of works, safeguarding the public interest typically takes precedence over compensating individual losses.

## Conclusions and implications

### Research findings

This study employed grounded theory and descriptive statistics to systematically analyze Chinese court judgments applying punitive damages in intellectual property infringement cases. It reached the following conclusions:

(1) Chinese judges assess the “seriousness” of intellectual property infringements by reference to 16 elements; for example, refusal to cease the infringement, repeated infringement, obstruction of evidence, and dishonest conduct. These elements can be summarized into three core categories: “Z1:subjective malevolence”, “Z2: degree of conduct”, and “Z3:resulting damage”. Together, they depict the infringement across its source (intent), process (conduct), and outcome (harm), and there exist pairwise causal mapping relationships among Z1, Z2, and Z3: that is, between Z1 and Z2, Z2 and Z3, and Z1 and Z3. In general, subjective malevolence is causally connected to more severe conduct, and more severe conduct is causally connected to more extensive damage; there is also a direct causal link from subjective malevolence to extensive damage (Z1 → Z3). However, these pairwise causal relationships are general tendencies rather than absolute rules, and specific cases may depart from them.
(2) Across different types of intellectual property cases, the factors that judges emphasize when applying punitive damages vary. In copyright infringement cases, judges primarily examine the infringer’s subjective malevolence. In trademark and patent infringement cases, they pay particular attention to the scale and duration of the infringement. In trade-secret cases, they focus on the relationship between the parties, as well as the losses suffered by the right holder.
(3) When awarding punitive damages, courts consider both the harm to the right holder and the damage to society. In cases involving factors that cause significant social harm, courts are much more likely to impose a higher punitive-damages multiplier.
(4) The application of punitive damages in intellectual property infringement cases is closely related to the three core purposes of punitive damages: deterrence, compensation, and safeguarding the public interest. Therefore, when applying punitive damages in intellectual property cases, it is necessary to consider not only the severity of the infringement but also these underlying purposes. Only in this way can we prevent judgment bias in applying punitive damages in intellectual property cases.

#### Research shortcomings and prospects.

Although comprehensiveness and integrity were considered as much as possible in data collection and coding, and the principle of theoretical saturation was followed, the textual dataset still has limitations. First, judicial decisions inevitably reflect a degree of judicial subjectivity. Second, China did not codify the comprehensive application of punitive damages in the field of intellectual property until the 2020 Civil Code, resulting in an insufficient number of IP cases in which punitive damages have been applied.

Methodologically, grounded theory can describe the key elements of the “seriousness” of intellectual property infringement and the logical relationships among them; however, it does not provide a quantitative model for the degree of “seriousness.”

Looking ahead, as the number of Chinese cases awarding punitive damages for intellectual property infringement increases, newly released judgments can be re-coded using open, axial, and selective coding. In addition, the Delphi method can be employed to further verify and supplement the key elements and the logic relating to serious circumstances in punitive damages for IP infringement. Alternatively, quantitative approaches can be used to develop and validate a scale for measuring serious circumstances, so that the punitive-damages regime can be implemented in the IP field in a more scientific, reasonable, precise, and rigorous manner.

## Supporting information

S1 Data Statistics of case information – en. (XLSX)

S2 Data Statistics of case information. (XLSX)

## References

[1] Rendleman D. Common law punitive damages: something for everyone. University of St Thomas Law Journal. 2009;7:2.

[2] Rustad M, Koenig T. The historical continuity of punitive damages awards: reforming the tort reformers. American University Law Review. 1993;42:1269.

[3] Ebert I. The power of punitive damages, is Europe missing out?. Journal of European Tort Law. 2013;4:95.

[4] National People’s Congress of the PR of C. The civil code of the People’s Republic of China. http://www.npc.gov.cn/c2/c30834/202006/t20200602_306457.html 2020. 2025 March 31.

[5] Li ZH. On the “Serious Circumstances” Requirement of Punitive Compensation for Intellectual Property Infringement from the Perspective of the Civil Code. Jinan Journal (Philosophy & Social Sciences). 2021;5:45–53.

[6] Vanleenhove C. The current European perspective on the exequatur of U.S. punitive damages: Opening the gate but keeping a guard. Polish Yearbook of International Law. 2015;35:235–64.

[7] Yuan XT. Justification basis and system construction of punitive compensation for intellectual property infringement. Gansu Social Sciences. 2014;5:196.

[8] Zhang P. The legitimacy and basic construction of intellectual property punitive compensation system. Intellectual Property. 2016;4:104–5.

[9] Goldberg JCP. The Constitutional Status of Tort Law: Due Process and the Right to a Law for the Redress of Wrongs. Yale Law Journal. 2005;115:524, 530.

[10] Polsky GD, Markel D. Taxing Punitive Damages. Virginia Law Review. 2010;96: 1295–7.

[11] Madden MS. Renegade Conduct and Punitive Damages in Tort. South Carolina Law Review. 2002;53:1190.

[12] Bush BJ. The Overlooked Function of Punitive Damages. Rutgers Law Journal. 2014;44:161–5.

[13] Reddington JA. To Caesar what is Caesar’s: an audacious claim for punitive damage reform in patent law. Liberty University Law Review. 2016;10:201–2.

[14] Guan YY. The analysis of the applicable conditions of punitive damages for infringement of intellectual property rights. Journal of Law Application. 2021;1:43–4.

[15] Su ZF. On the Goal, Orientation and Judicial Application of Punitive Compensation System of Intellectual Property in China. China Journal of Applied Jurisprudence. 2021;1:132–45.

[16] Koziol H. Punitive damages: Admission into the seventh legal heaven or eternal damnation?. Punitive Damages: Common Law and Civil Law Perspectives. Vienna: Springer-Verlag. 2009. 275–308.

[17] Zhu D. Economic analysis of punitive compensation system of intellectual property rights. Oriental Law. 2014;6:49–58.

[18] Rustad ML. The Supreme Court and Me: Trapped in Time with Punitive Damages. Widener Law Journal. 2008;17:783, 785.

[19] Seymour v. McCormick, 57 U.S. (16How.) 1853;480.

[20] Lam, Inc., 718 F.2d at.747.

[21] In re Seagate Tech., LLC, 497 F.3d 1360 (Fed Cir. 2007).

[22] Stryker Corp. v. Zimmer, Inc., 782 F.3d 649 (Fed Cir. 2014).

[23] Halo Elecs., Inc. v. Pulse Elecs., Inc., 769 F.3d 1371(Fed Cir. 2014).

[24] Read Corp. v. Portec, Inc., 970 F 2d 816, 826-828 (Fed Cir 1992).

[25] Street H. Principle of the law of damages. London: Sweet & Maxwell. 1962.

[26] Clearview Plumbing & Heatings Ltd. v. Clockwork IP, LLC, Ottawa, Ontario, February 13, 2018.

[27] Judical protection for intellectual property. Analysis of punitive damages for foreign intellectual property infringement and foreign rule of law. https://www.chinaiprlaw.cn/index.php?id=1233 2015. 2025 March 31.

[28] Wang CM, Wang R. Judgment of “Serious Circumstance” in Punitive Damages for Intellectual Property: Based on the Research Approach of Dynamic System Theory. Legal Forum. 2022;2:143–4.

[29] He YD. Approaches to properly interpreting the “serious circumstances” requirement of punitive damages for intellectual property rights infringement. Jinan Journal (Philosophy & Social Sciences). 2023;7:30–44.

[30] BMW of North America, Inc. v. Gore. U.S. 1996. 559.

[31] Cooper Industries, Inc. v. Leatherman Tool Group, Inc. U.S. 2001. 42.

[32] Whiten v. Pilot Insurance Co. SCC. 2002. paras. 94-95, 112-125.

[33] Chen ZB, Huang S. On the determination of the base and multiple of punitive compensation for intellectual property infringement. Social Sciences in Guangxi. 2022;5:123, 126.

[34] Ding WY, Zhang LL. Research on Judicial Determination of Punitive Compensation for Intellectual Property Infringement. Intellectual Property. 2021;2:72.

[35] Xu CM, Yang HH. Establishment of Rules for Determining Multiple of Intellectual Property Punitive Damages. Science Technology and Law (Chinese-English). 2023;3:103–5.

[36] Ni ZL. The application of the principle of proportionality in the quantification of punitive damages for intellectual property rights. Intellectual Property. 2021;7:24–38.

[37] Wang Z. Compensation for Damages. Beijing: Peking University Press. 2017.

[38] Liu YL. Comparative analysis of the IP punitive damages systems and its inspiration. Law Science. 2022;7:131–48.

[39] Glaser BG, Strauss AL. The discovery of grounded theory: Strategies for qualitative research. New York: Adline de Gruyter. 1967.

[40] IPHouse Database. https://www.iphouse.cn/

[41] The minimal data set. 10.6084/m9.figshare.30461615

[42] Kametz K. Constructing grounded theory: A practical guide to qualitative research. Chongqing: Chongqing University Press. 2009.

[43] Strauss AL. Qualitative Analysis for Social Scientists. New York: Cambridge University Press. 1987.

[44] Holton JA, Walsh I. Classic grounded theory: applications with qualitative and quantitative data. Beijing: Peking University Press. 2021.

[45] Holzman LA, Mendelsohn M. Punitive damages under the copyright act. IPL Newsletter. 2005;23:21–42.

[46] Gong XY, Liu C. Deconstruction and path exploration of the application of punitive compensation for intellectual property rights: Taking Shanghai’s first case of punitive damages for intellectual property rights as a research example. Journal of Law Application. 2020;24:152–3.

[47] The Subject Group of Futian District People’s Court of Shenzhen City, Guangdong Province. System construction of punitive damages for trademark infringement. Intellectual Property. 2020;5:40–54.

[48] Rookes v Barnard. UKHL. 1964. 38.

[49] Richard v Allstate Ins Co. Federal Reporter. 502.

[50] Sharkey CM. Punitive Damages as Societal Damages. The Yale Law Journal. 2003;113(2):347. doi: 10.2307/3657525

[51] Guangzhou Intellectual Property Court. Case No. 2442 civil appeal judgment, Guangdong Province. 2020.

[52] Guangdong High People’s Court. Case No. 147 civil retrial judgment. 2019.

[53] Supreme People’s Court of China. Case No. 816 IP civil final judgment. 2021.

[54] Whiten v Pilot Insurance Co. 2002.

[55] Wu HD. The private law foundations and judicial application of punitive damages in intellectual property. Law Review. 2021;3:26–7.

